# A genome-wide phylogeny of jumping spiders (Araneae, Salticidae), using anchored hybrid enrichment

**DOI:** 10.3897/zookeys.695.13852

**Published:** 2017-09-04

**Authors:** Wayne P. Maddison, Samuel C. Evans, Chris A. Hamilton, Jason E. Bond, Alan R. Lemmon, Emily Moriarty Lemmon

**Affiliations:** 1 Department of Zoology, University of British Columbia, 6270 University Boulevard, Vancouver, British Columbia, V6T 1Z4, Canada; 2 Department of Botany and Beaty Biodiversity Museum, University of British Columbia, 6270 University Boulevard, Vancouver, British Columbia, V6T 1Z4, Canada; 3 Department of Biological Sciences, Auburn University, Auburn, AL, USA; 4 Auburn University Museum of Natural History, Auburn University, Auburn, AL, USA; 5 Florida Museum of Natural History, University of Florida, 3215 Hull Rd, Gainesville, FL, 32611; 6 Department of Scientific Computing, Florida State University, Tallahassee, FL, USA; 7 Department of Biological Science, Florida State University, Tallahassee, FL, USA

**Keywords:** Dionycha, jumping spiders, salticids, systematics, phylogenomics

## Abstract

We present the first genome-wide molecular phylogeny of jumping spiders (Araneae: Salticidae), inferred from Anchored Hybrid Enrichment (AHE) sequence data. From 12 outgroups plus 34 salticid taxa representing all but one subfamily and most major groups recognized in previous work, we obtained 447 loci totalling 96,946 aligned nucleotide sites. Our analyses using concatenated likelihood, parsimony, and coalescent methods (ASTRAL and SVDQuartets) strongly confirm most previous results, resolving as monophyletic the Spartaeinae, Salticinae (with the hisponines sister), Salticoida, Amycoida, Saltafresia, and Simonida. The agoriines, previously difficult to place beyond subfamily, are finally placed confidently within the saltafresians as relatives of the chrysillines and hasariines. Relationships among the baviines, astioids, marpissoids, and saltafresians remain uncertain, though our analyses tentatively conclude the first three form a clade together. Deep relationships, among the seven subfamilies, appear to be largely resolved, with spartaeines, lyssomanines, and asemoneines forming a clade. In most analyses, *Onomastus* (representing the onomastines) is strongly supported as sister to the hisponines plus salticines. Overall, the much-improved resolution of many deep relationships despite a relatively sparse taxon sample suggests AHE is a promising technique for salticid phylogenetics.

## Introduction

Understanding the relationships of jumping spiders (Salticidae) long posed a challenge, given their diversity in forms and species (about 6,000 described, World Spider Catalog 2017). Recent data from a handful of sequenced genes has, however, begun to resolve many aspects of the group’s broad phylogenetic structure (Maddison and Hedin 2003, Bodner and Maddison 2012, Maddison et al. 2014). Combined with morphological information, these results have led to a comprehensive phylogenetic classification (Maddison 2015) and are beginning to enable inferences about evolutionary patterns in salticids’ structures, ecology, and behaviour. Two major gaps in knowledge remain to be filled, however, before the phylogeny can provide a high-resolution lens on salticid evolution. First, the great majority of known species are unstudied phylogenetically (and many others undiscovered taxonomically), and therefore few details are available about shallower phylogeny in most tribes and genera of the family. Second, the few genes studied do not give definitive answers in several key areas of the deeper parts of the phylogeny. Maddison et al. (2014) were unable to resolve the relationships among the seven subfamilies (as defined by Maddison 2015), except for the sister group relationship between Hisponinae and Salticinae. They were also unable to place the peculiar agoriines, and to determine the relationships among the baviines, Marpissoida, Astioida, and Saltafresia; support for the Saltafresia and Simonida was only tentative.

Our goal here is to answer remaining questions about broad salticid relationships, using data from across the genome. An efficient method to obtain data on hundreds of genes is Anchored Hybrid Enrichment (AHE; Lemmon et al. 2012; Lemmon and Lemmon 2013), a high-throughput genomics technique that uses probes designed for highly conserved DNA regions flanked by less-conserved regions. AHE has been applied for both deep and shallow relationships in spiders (Hamilton et al. 2016a,b), where it shows considerable promise for resolving phylogeny based on genome-wide data. We here apply AHE to salticids, using a combination of Spider Probe Kit versions 1 and 2 designed for spiders by Hamilton et al. (2016b, unpublished). The AHE Spider Probe Kit targets 585 phylogenetically-informative loci across the Order Araneae and delivers phylogenetic utility at both deep and shallow taxonomic depths. By providing a set of molecular markers that can be used to address evolutionary questions at multiple hierarchical levels, as well as across different research groups, the AHE Spider Probe Kit is being used to answer larger questions about spider phylogeny and evolution (Hamilton et al. 2016a,b).

## Methods

### Taxon sampling

Specimens sampled are listed in Table 1, representing 33 salticid genera belonging to 26 tribes and 6 subfamilies among the 30 tribes and 7 subfamilies currently recognized in the Salticidae (Maddison 2015). The one subfamily not sampled is the Eupoinae; the four tribes not represented are the amycoid tribe Huriini and the astioid tribes Neonini, Mopsini, and Viciriini. In addition, 12 dionychan outgroups are included, representing families inferred as more and less closely related to salticids by Wheeler et al. (2017). *Homalonychus* is used as the most distant outgroup.

When multiple specimens from a single genus (e.g. two *Hasarius*) were sampled, their DNA was pooled and they were treated as a single terminal taxon in analyses, resulting in 34 salticid and 12 outgroup terminal taxa (see “+” symbols in Table 1). This was done in an attempt to obtain our target DNA quantity of 500ng for sequencing. The one exception to this is *Sarinda*, whose DNA extraction and sequencing was done separately for two separate species. The specimens pooled for a terminal taxon appear to represent the same species in all cases but three. For *Agorius*, *Fluda*, and *Tisaniba*, two species were pooled for each (see Table 1), and thus those terminal taxa are chimeric. There is no doubt, based on morphology, that the two *Agorius* are sisters among the species included here, and likewise for the two *Fluda* and the two *Tisaniba*.

Voucher specimens are preserved in the Spencer Entomological Collection of the Beaty Biodiversity Museum (vouchers whose IDs in Table 1 start with “SCE”) and in the Auburn University Museum of Natural History (AUMNH) (vouchers with other IDs).

### DNA extraction, sequencing, filtering, and alignment

Specimens were preserved in 95% ethanol, and stored between two months and 10 years before use. DNA extractions were done using the Qiagen DNEazy blood and tissue kit, using the protocol for <10 mg samples. The second through fourth pairs of legs were used if they provided sufficient sample volume; otherwise, the carapace and sometimes the distal part of the abdomen was added.

Library preparation, enrichment, and sequencing were conducted at the Center for Anchored Phylogenomics at Florida State University (http://www.anchoredphylogeny.org). After extraction, up to 500ng of each DNA sample was sonicated to a fragment size of ~300–800 bp using a Covaris E220 ultrasonicator. Indexed libraries were then prepared following Meyer and Kircher (2010), but with modifications for automation on a Beckman-Coulter Biomek FXp liquid-handling robot (see Hamilton et al. 2016b for details). Size-selection was performed after blunt-end repair using SPRI select beads (Beckman-Coulter Inc.; 0.9x ratio of bead to sample volume). Indexed samples were pooled at equal quantities (16 samples per pool), and then each pool was enriched using the AHE Spider Probe kit v1 developed by Hamilton et al. (2016b) and a modified v2 (Hamilton et al. unpublished), which has been refined to yield greater enrichment within araneomorph spiders than the original version. After enrichment, the two enrichment reactions were pooled in equal quantities and sequenced on one PE150 Illumina HiSeq 2500 lanes at Florida State University Translational Science Laboratory in the College of Medicine.

**Table 1.** Specimens from which Anchored Hybrid Enrichment data were obtained. A “+” at the start of a row indicates that that specimen’s DNA was combined with that of the previous specimen for sequencing, to yield a single analyzed terminal taxon.

Species	Voucher ID	Sex	Locality	Latitude - longitude
**Salticidae**				
Agorius aff. borneensis Edmunds & Proszynski, 2001	SCE0002	m	Malaysia: Sarawak: Mulu Nat. Pk.	4.05°N 114.86°E
+ *Agorius* sp.	SCE0035	f	Malaysia: Sarawak: Mulu Nat. Pk.	4.05°N 114.86°E
*Asemonea sichuanensis* Song & Chai, 1992	SCE0016	m	China: Guangxi: Ningming County	21.811°N 107.217°E
*Bavia aericeps* Simon, 1877	SCE0008	m	Papua New Guinea: S. Highlands Prov.: Tualapa	5.283°S 142.498°E
*Breda apicalis* Simon, 1901	SCE0022	m	Ecuador: Orellana: Yasuní Res. Stn.	0.677°S 76.402°W
*Carrhotus sannio* (Thorell, 1877)	SCE0044	m	China: Guangxi: Ningming County	21.822°N 107.029°E
*Cocalodes macellus* (Thorell, 1878)	SCE0005	m	Papua New Guinea: S. Highlands Prov.: Putuwé	5.231°S 142.532°E
*Colonus sylvanus* (Hentz, 1846)	SCE0015	m	U.S.A.: Mississippi: Holmes County State Park	33.031°N 89.928°W
Fluda cf. usta Mello-Leitão, 1940	SCE0009	m	Ecuador: Orellana: Río Bigal Reserve	0.53°S 77.418°W
+ *Fluda elata* Galiano, 1986	SCE0057	m	Ecuador: Orellana: SE of Río Bigal Reserve	0.53-5°S 77.42°W
*Freya decorata* (C. L. Koch, 1846)	SCE0012	m	Ecuador: Orellana: Yasuní Res. Stn.	0.674°S 76.397°W
*Habronattus ophrys* Griswold, 1987	SCE0065	m	Canada: British Columbia: Furry Creek	49.581°N 123.208°W
*Harmochirus brachiatus* (Thorell, 1877)	SCE0029	m	Malaysia: Sarawak: Kubah Nat. Pk.	1.605-6°N 110.185-7°E
*Hasarius adansoni* (Audouin, 1825)	SCE0011	m	Singapore: Labrador Park	1.27°N 103.80°E
+ *Hasarius adansoni* (Audouin, 1825)	SCE0049	m	China: Guangxi: Tianlin County	24.46°N 106.37°E
*Heliophanus lineiventris* Simon, 1868	SCE0039	m	Spain: Albacete: Villa de Chinchilla	38.9143°N 01.4618°W
*Idastrandia* sp.	SCE0031	f	Malaysia: Sarawak: Mulu Nat. Pk.	4.05°N 114.86°E
*Lapsias canandea* Maddison, 2012	SCE0020	m	Ecuador: Esmeraldas: Reserva Canandé	0.5167°N 79.1934°W
*Leikung porosa* (Wanless, 1978)	SCE0003	m	Malaysia: Sarawak: Lambir Hills Nat. Pk.	4.197-8°N 114.040°E
+ *Leikung* sp.	SCE0058	f	Malaysia: Sarawak: Lambir Hills Nat. Pk.	4.203°N 114.028-9°E
+ *Leikung* sp.	SCE0059	f	Malaysia: Sarawak: Lambir Hills Nat. Pk.	4.2025°N 114.0308°E
*Lyssomanes viridis* (Walckenaer, 1837)	SCE0018	m	U.S.A.: Mississippi: Wall Doxey State Park	34.665°N 89.466°W
*Mintonia ramipalpis* (Thorell, 1890)	SCE0021	m	Malaysia: Sarawak: Mulu Nat. Pk.	4.038°N 114.813°E
*Myrmarachne* sp.	SCE0053,4	m	Malaysia: Pahang: Tanah Rata	4.46°N 101.40°E
*Naphrys pulex* (Hentz, 1846)	SCE0038	m	Canada: Muskoka Dist.: Port Cunnington	45.259°N 79.026°W
*Noegus* sp.	SCE0023	m	Ecuador: Orellana: Yasuní Res. Stn.	0.68°S 76.39°W
*Onomastus* sp.	SCE0047	f	China: Guangxi: Fangchenggang City	21.683°N 107.649°E
+ *Onomastus* sp.	SCE0048	f	China: Guangxi: Ningming County	21.815°N 107.305°E
Orthrus aff. muluensis Wanless, 1980	SCE0040	f	Malaysia: Sarawak: Lambir Hills Nat. Pk.	4.199°N 114.037°E
+ Orthrus aff. muluensis Wanless, 1980	SCE0041	f	Malaysia: Sarawak: Lambir Hills Nat. Pk.	4.202°N 114.042°E
*Phidippus johnsoni* (Peckham & Peckham, 1883)	SCE0024	m	U.S.A.: Oregon: Mt. Hebo	45.214°N 123.755°W
*Salticus scenicus* (Clerck, 1757)	SCE0045	m	Canada: British Columbia: Kelowna	49.95°N 119.401°W
*Sarinda hentzi* (Banks, 1913)	AUMS16070	m	U.S.A.: Alabama: Elmore Co.	32.52265°N 86.0024°W
*Sarinda* sp.	SCE0046	m	Ecuador: Napo: Estación Biológica Jatun Sacha	1.067°S 77.617°W
*Sassacus* sp.	AUMS16722	f	U.S.A.: Washington: Northcreek	46.8908°N 123.1967°W
*Scopocira cyrili* Costa & Ruiz, 2014	SCE0060,1,2	m	French Guiana: Commune Règina, les Nourages	4.0691°N 52.6689°W
*Sitticus fasciger* (Simon, 1880)	SCE0028	m	Canada: Ontario: Burlington	43.3507°N 79.7593°W
*Tisaniba bijibijan* Zhang & Maddison, 2014	SCE0050	f	Malaysia: Sarawak: Lambir Hills Nat. Pk.	4.200°N 114.036°E
+ *Tisaniba dik* Zhang & Maddison, 2014	SCE0051	f	Malaysia: Sarawak: Mulu Nat. Pk.	4.0380°N 114.8137°E
+ *Tisaniba dik* Zhang & Maddison, 2014	SCE0052	f	Malaysia: Sarawak: Mulu Nat. Pk.	4.0380°N 114.8137°E
*Titanattus* sp.	SCE0055,6	f	Brazil: Pará: Algodoal	0.580°S 47.586°W
*Tomomingi* sp.	SCE0017	f	Gabon: Woleu-Ntem:Tchimbélé	0.629°N 10.404°E
*Yllenus arenarius* Simon, 1868	SCE0042,3	j	Poland: Kozki	52.361°N 22.870°E
**Outgroups**				
Clubionidae: *Clubiona* sp.	G1765	f	U.S.A.: California: Torrey Pines S.P.	32.92799°N 117.2575°W
Ctenidae: *Ctenus exlineae* Peck, 1981	G1699	f	U.S.A.: Arkansas: Stone Co., S. Calico Rock	35.9952°N 92.12200°W
Eutichuridae: *Cheiracanthium* sp.	G1048	m	U.S.A.: California: San Diego Co., Lake Murray	32.7862°N 117.0360°W
Gnaphosidae: *Zelotes* sp.	AUMS16708	f	U.S.A.: Washington: Stella	46.2614°N 123.1317°W
Homalonychidae: *Homalonychus theologus* Chamberlin, 1924	AUMS11918	f	U.S.A.: California: Imperial Co.	—
Lycosidae: *Alopecosa* sp.	AUMS16717	—	U.S.A.: Washington: Bear Canyon	46.71194°N 120.8906°W
Lycosidae: *Schizocosa saltatrix* (Hentz, 1844)	AUMS19518	—	U.S.A.: Tennessee: Heck Hollow Road	36.3319°N 82.9552°W
Miturgidae: *Zora spinimana* (Sundevall, 1833)	ARA0192	f	Switzerland: Grison Alps, Alp Flix, Salatinas:	46.5131°N 9.6430°E
Oxyopidae: *Oxyopes* sp.	AUMS16731	m	U.S.A.: Alabama: Lee Co., Auburn	32.5820°N 85.4228°W
Philodromidae: *Philodromus barrowsi* Gertsch, 1934	SCE0063	m	U.S.A.: Arizona: Tumacacori	31.562°N 111.046°W
Thomisidae: *Coriarachne* sp.	AUMS16723	f	U.S.A.: Washington: Little Rock Road	46.8728°N 123.0239°W
Zoropsidae: *Zoropsis spinimana* (Dufour, 1820)	ARA1365	f	Slovenia: Ljubljana	46.0485°N 14.5079°E

Prior to assembly, overlapping paired reads were merged following Rokyta et al. (2012). For each read pair, the probability of obtaining the observed number of matches by chance was evaluated for each possible degree of overlap. The overlap with the lowest probability was chosen if the p-value was less than 10^-10^, a stringent threshold that helps avoids chance matches in repetitive regions (see Rokyta et al. 2012 for details). Read pairs failing to merge were utilized but left unmerged during the assembly.

Divergent reference assembly was used to map reads to the probe regions and extend the assembly into the flanking regions (see Prum et al. 2015 and Hamilton et al. 2016b for details). For this analysis, the *Aphonopelma*, *Aliatypus*, *Ixodes* and *Hypochilus* references (Hamilton et al. 2016b) were utilized as references. Preliminary matches were called if at least 17 of 20 spaced-kmer bases matched and the preliminary matches were confirmed if at least 55 of 100 consecutive bases matched. Assembly contigs derived from less than 23 reads were removed in order to reduce the effects of cross contamination and rare sequencing errors in index reads.

Orthology was determined among the homologous consensus sequences at each locus following Prum et al. (2015) and Hamilton et al. (2016b). Pairwise distances among homologs were computed for each locus based on the percent of shared continuous and spaced 20-mers. Sequences were clustered using a Neighbor-Joining algorithm by distance, but allowing at most one sequence per species to be in a given cluster. In order to reduce the effects of missing data, data were reduced by removing from downstream processing clusters that contained fewer than 50% of the species. The result of this assessment was 492 orthologous clusters (loci).

For all samples except *Tisaniba*, the nHomologs statistic presented in the Supplementary Table shows value near 1, indicating that at each locus approximately one homolog was recovered by the assembler. This is an indication that recent gene duplication and loss is very low in this group, and that our results are not compromised by the deep arachnid whole-genome duplication (Schwager et al. 2017). It also indicates that the individuals whose DNA was pooled for each species were quite similar (the assembler interpreted any differences at the level of allelic differences). This is not the case for *Tisaniba*, which had an elevated nHomolog value of 1.71, meaning that at 71% of the loci, two homologs were identified and separated into different consensus sequences. For these loci the orthology method would choose the consensus sequence most similar to that of the most similar relatives, and likely removed the other consensus from downstream analysis.

Sequences in each orthologous cluster were aligned using MAFFT v7.023b (Katoh and Standley 2013), using the --genafpair and --maxiterate 1000 flags. The alignment for each locus was then trimmed/masked using the steps described in Hamilton et al. (2016b). Each alignment site was identified as “conserved” if the most commonly observed character was present in > 50% of the sequences. Each sequence was scanned for regions that did not contain at least 10 of 20 characters matching to the common base at the corresponding conserved site. Characters from regions not meeting this requirement were masked. Third, sites with fewer than 23 unmasked bases were removed from the alignment. Geneious version 7 (www.geneious.com; Kearse et al. 2012) was used to visually inspect each masked alignment and to remove regions of sequences identified as obviously misaligned or paralogous. Trimming resulted in some loci being deleted, yielding a final total of 447 loci. This represents a higher success rate than Hamilton et al. (2016), This represents a higher success rate than Hamilton et al. (2016), whose study had greater breath, across all spiders, and used an older probe set.

In preparation for phylogenetic analyses, the 447 trimmed AHE loci were re-aligned individually with MAFFT version 7.058b (Katoh and Standley 2013) using the L-INS-i option (--localpair --maxiterate 1000). Although assigning codon positions could have allowed better model partitioning in the phylogenetic analysis, we were unable to do so because the loci are often relatively short (average about 560 bases; see Supplementary Table) and we lack a well-annotated reference transcriptome. Our attempts to assign codon positions via TransDecoder version 3.0.1 (Haas et al. 2013) yielded unrealistic results for many loci, and so we left codon positions unassigned.

### Phylogenetic analyses

We inferred the phylogeny for the 46 taxa using Maximum Likelihood, parsimony, and SVDQuartets applied to a concatenated supermatrix of the 447 aligned loci, and using ASTRAL (a coalescent-based approach, like SVDQuartets) applied to ML-reconstructed gene trees of the 447 separate loci.

Two Maximum Likelihood (ML) analyses on the concatenated matrix were performed using RAxML version 8.2.8 (Stamatakis 2014). One left the matrix unpartitioned. The other used partitions chosen by PartitionFinder version 1.1.1 (Lanfear et al. 2012) based on an initial partition by locus. PartitionFinder grouped the loci via a relaxed clustering algorithm assuming linked branch lengths and evaluating 10% of schemes at each step according to BIC score. We used relaxed clustering as, for large datasets such as ours, it has been demonstrated to produce results consistently comparable to a greedy algorithm but with much more computational efficiency (Lanfear et al. 2014). The best scheme according to our PartitionFinder analyses grouped loci into 21 partitions. Both maximum likelihood analyses assumed the GTR+gamma+I model.

We present as our primary result the best-scoring ML tree from the partitioned supermatrix and 200 search replicates. Robustness of clade support was explored by a bootstrap analysis with 1000 replicates, in each of which 5 search replicates were done.

Parsimony bootstrap analysis was performed by PAUP* version 4.0a151 (Swofford 2002), with 1000 replicates, for each of which we used TBR branch rearrangement, multrees, maxtrees = 100, and 2 search replicates.

We also used two methods based on the multi-species coalescent model to infer the species phylogeny, SVDQuartets (Chifman and Kubatko 2015) and ASTRAL II (Mirarab et al. 2014). SVDQuartets was performed by PAUP* version 4.0a150 using exhaustive quartet sampling and 1000 bootstrap replicates. The ASTRAL analysis was performed by version 4.7.12 using default settings, based on the 447 gene trees, one from each locus, obtained by RAxML version 8.2.8 from a simple ML search (model GTRGAMMA, unpartitioned).

## Results

Hybrid enrichment results are shown in the Supplementary Table. The 447 loci obtained in the final filtered data set represent for most taxa about 80 kb of nucleotide sequence. We were less successful at obtaining data for two taxa, with *Schizocosa saltatrix* having only 9377 nucleotides sequenced, and *Yllenus arenarius* having 36069 nucleotides. The “on target” percentage of *Yllenus* was low, suggesting either that its genome is unusually large, or that the sample included also some non-spider DNA. The other taxa had between 76,262 (*Clubiona*) and 91,238 (*Hasarius adansoni*) nucleotides sequenced. Alignments for each of the 477 loci are deposited, along with phylogenetic results, to Dryad (http://dx.doi.org/10.5061/dryad.n2b3h).

**Figure 1.**
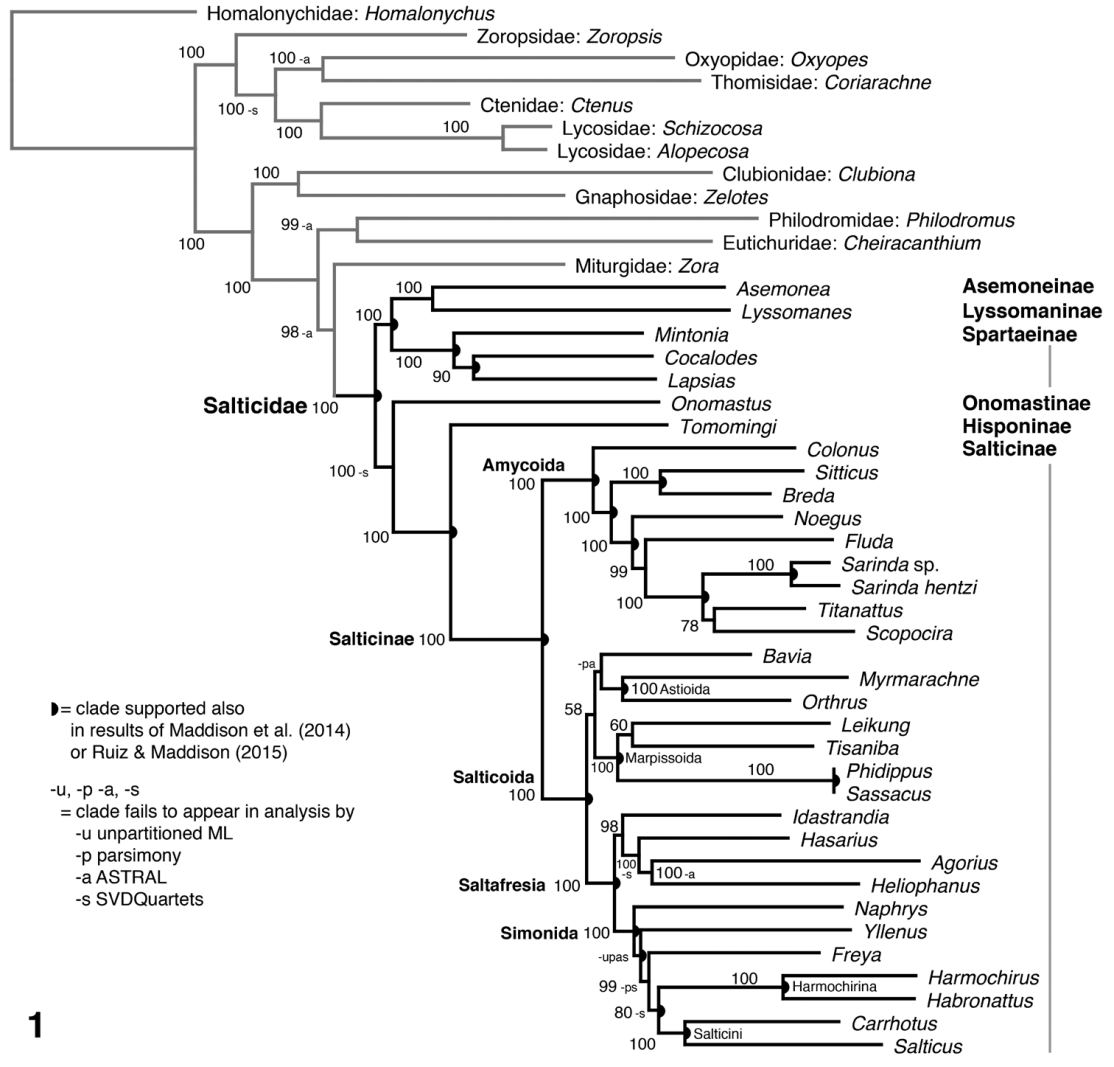
Maximum likelihood phylogeny from the partitioned concatenated matrix of 447 loci captured by Anchored Hybrid Enrichment. Numbers indicate percentage of likelihood bootstrap replicates showing the clade. Half circle indicates clades supported also in the results of Maddison et al. (2014) or, for the Amycoida, of Ruiz and Maddison (2015). Letters u, p, a, and s indicate clades that fail to appear in the analyses by unpartitioned likelihood, parsimony, ASTRAL and SVDQuartets respectively.

Fig. 1 shows the ML tree from the partitioned concatenated supermatrix. Bootstrap values are high for most clades. The unpartitioned ML, parsimony, ASTRAL and SVDQuartets gave largely concordant results, differing only where marked in Fig. 1 by -u, -p, -a, and -s respectively. In particular, unpartitioned ML places *Yllenus* as the sister to the rest of the Simonida (though with low bootstrap support); parsimony places *Yllenus* and *Naphrys* as sisters, and Freya as sister to *Harmochirus* and *Habronattus*; ASTRAL places *Bavia* as sister to the astioids and marpissoids, and *Yllenus* as the sister to the rest of the Simonida; SVDQuartets trades the positions of *Idastrandia* and *Hasarius* and rearranges the Simonida.

## Discussion

This first genome-wide analysis of salticids resolves the group’s phylogeny with greater confidence than previous studies, confirming and extending those results based on far fewer genes (Maddison et al. 2014; Ruiz and Maddison 2015; Maddison 2015). The results corroborate the monophyly of the Salticinae, a major clade with more than 90% of described salticid species, including most familiar species. The Spartaeinae, which includes the well-known *Portia*, is also supported (in our analysis: *Mintonia*, *Cocalodes*, *Lapsias*). Major clades corroborated within the Salticinae are the Salticoida (*sensu* Maddison 2015), Saltafresia, Simonida, Amycoida, and Marpissoida (here: *Leikung*, *Tisaniba*, *Phidippus*, *Sassacus*). Other clades consistent with the previous results of Maddison et al. (2014, 8 genes, salticid-wide) and Ruiz and Maddison (2015, 5 genes, within the Amycoida) are indicated with semicircles on Fig. 1.

The relationships among the subfamilies, previously poorly resolved (Maddison et al. 2014), are strongly supported in our analyses. Unsurprising is the relationship between the Hisponinae and Salticinae, which has been supported by both molecular and morphological data (Maddison 2015). The relationship among asemoneines, lyssomanines and spartaeines was anticipated (Maddison et al. 2014) but not previously well supported.

A novel result is the placement of Onomastinae as sister to Hisponinae plus Salticinae. Onomastines, like the lyssomanines and asemoneines, are long-legged translucent spiders with complex palpi and an ocular area relatively small compared to other salticids (see Wanless 1980). The distinctive features of onomastines, lyssomanines and asemoneines might have been interpreted as ancestral for the family, or as synapomorphies uniting them (Maddison 2015). Their separate placement here suggests that either their form is convergent, or that the more familiar compact brown body with an expanded ocular area evolved independently in spartaeines and hisponines+salticines. We do note, however, that despite the 100% ML bootstrap support for onomastines+hisponines+salticines, not all analyses agree on this placement. The SVDQuartets analysis places *Onomastus* as sister to *Asemonea*+*Lyssomanes*+Spartaeinae, as also recovered from 8 genes by Maddison et al. (2014).

Within the Salticinae, our data have succeeded in resolving the placement of one puzzling group, the agoriines, whose position was problematic to Maddison et al. (2014). Our 447 locus data clearly supports placing the agoriines within the Saltafresia, in a group with chrysillines (here represented by *Heliophanus*) and hasariines. Most analyses place *Agorius* sister to *Heliophanus*, though ASTRAL places it with the nearby *Hasarius*. Maddison et al. (2014) found *Agorius* and its close relative *Synagelides* to have unstable placement, on long branches, and varying in position drastically among the different analyses. Interestingly, their All Genes salticine analysis (their figure 18) placed agoriines with the chrysillines, a placement strongly supported in our analyses. Maddison (2015) notes the similarities of the genitalia of agoriines with the two groups indicated as close relatives here, the chrysillines and hasariines.

The relationships among the four major subgroups of Salticoida (*sensu* Maddison 2015) — Marpissoida, Astioida, Baviini, and Saltafresia — were not resolved well by Maddison et al. (2014: 80). Bodner and Maddison (2012) suggested the first three form a clade, but this was not corroborated by the results of Maddison et al. (2014). Our data give support to Bodner and Maddison’s conclusion, though weakly. All analyses place *Bavia* in a clade with the Marpissoida and Astioida (together forming the sister group to the Saltafresia), but bootstrap support is only 58% for likelihood, 67% for parsimony, and 100% for SVDQuartets. The weak support for this clade may indicate a rapid early radiation of the Salticoida, and may require considerably more data to corroborate or refute. Within the tentative clade of Baviini+Marpissoida+Astioida the detailed relationships are unresolved. Likelihood and SVDQuartets place *Bavia* with the astioids *Myrmarachne* and *Orthrus* but with bootstrap support less than 50% for ML, 56% for SVDQuartets; parsimony places *Bavia* as sister to the Marpissoida; ASTRAL places *Bavia* as sister to Marpissoida+Astioida.

Within the Simonida, the Harmochirina (*Harmochirus*, *Habronattus*) and Salticini (*Carrhotus*, *Salticus*) are confirmed each as monophyletic and as sister lineages, as per Maddison et al. (2014). Deeper relationships in the Simonida, among the tribes, are unclear and vary by analysis. As shown in Fig. 1, likelihood recovers (*Naphrys*, (*Yllenus*, (*Freya*, (harmochirines, salticines)))), with *Naphrys* representing the Euophryini, *Yllenus* the Leptorchestini, and *Freya* the Aelurillini. However, ASTRAL obtains (*Y*,(*N*,(*F*,(h,s)))), SVDQuartets (*Y*,(h,(*N*,(*F*,s)))), and parsimony ((*Y*,*N*),((*F*,h),s)). A contributing factor to this poor resolution could be the poor sequence capture for *Yllenus*.

Given the strength of this broad data set and its concordance with previous results, we can now be reasonably confident in our current phylogenetic classification (Maddison 2015). Our results highlight what is needed for further progress. For the deeper parts of the phylogeny, most urgent is to include the Eupoinae, not only to determine their (currently ambiguous) placement (Maddison et al. 2014), but also because their inclusion would provide a test of the supported relationships among the subfamilies. Within the Salticinae, the most basic outstanding question concerns the relative relationships among baviines, astioids, marpissoids and saltafresians. To resolve this, a much larger fraction of the genome may be needed. Of course, even once our understanding of these broad relationships stabilizes, the bulk of salticid phylogeny remains still unresolved, as not only is there no explicit phylogenetic work on most of the described species, but many species remain to be discovered.

## Supplementary materials

Supplementary material 1 Supplementary table of assembly statistics Data type: Microsoft Excel Worksheet (.xlsx) Explanation note: Statistics describing raw reads, loci, sequence lengths, and other aspects of sequencing assembly for each of the 34 salticid taxa and 12 outgroup taxa. This dataset is made available under the Open Database License (http://opendatacommons.org/licenses/odbl/1.0/). The Open Database License (ODbL) is a license agreement intended to allow users to freely share, modify, and use this Dataset while maintaining this same freedom for others, provided that the original source and author(s) are credited.Wayne P. Maddison, Samuel C. Evans, Chris A. Hamilton, Jason E. Bond, Alan R. Lemmon, Emily Moriarty Lemmon
